# Development of Circumventricular Organs in the Mirror of Zebrafish Enhancer-Trap Transgenics

**DOI:** 10.3389/fnana.2017.00114

**Published:** 2017-12-07

**Authors:** Marta García-Lecea, Evgeny Gasanov, Justyna Jedrychowska, Igor Kondrychyn, Cathleen Teh, May-Su You, Vladimir Korzh

**Affiliations:** ^1^Institute of Molecular and Cell Biology, Agency for Science, Technology and Research, Singapore, Singapore; ^2^Department of Basic Biomedical Sciences, Universidad Europea de Madrid, Madrid, Spain; ^3^International Institute of Molecular and Cell Biology in Warsaw, Warsaw, Poland; ^4^Postgraduate School of Molecular Medicine, Medical University of Warsaw, Warsaw, Poland; ^5^RIKEN Center for Developmental Biology, Kobe, Japan; ^6^National Health Research Institutes (NHRI), Zhunan, Taiwan

**Keywords:** organum vasculosum laminae terminalis, subfornical organ, area postrema, median eminence, paraventricular organ, subcommissural organ, pineal-parapineal complex, choroid plexus

## Abstract

The circumventricular organs (CVOs) are small structures lining the cavities of brain ventricular system. They are associated with the semitransparent regions of the blood-brain barrier (BBB). Hence it is thought that CVOs mediate biochemical signaling and cell exchange between the brain and systemic blood. Their classification is still controversial and development not fully understood largely due to an absence of tissue-specific molecular markers. In a search for molecular determinants of CVOs we studied the green fluorescent protein (GFP) expression pattern in several zebrafish enhancer trap transgenics including Gateways (ET33-E20) that has been instrumental in defining the development of choroid plexus. In Gateways the GFP is expressed in regions of the developing brain outside the choroid plexus, which remain to be characterized. The neuroanatomical and histological analysis suggested that some previously unassigned domains of GFP expression may correspond to at least six other CVOs–the organum vasculosum laminae terminalis (OVLT), subfornical organ (SFO), paraventricular organ (PVO), pineal (epiphysis), area postrema (AP) and median eminence (ME). Two other CVOs, parapineal and subcommissural organ (SCO) were detected in other enhancer-trap transgenics. Hence enhancer-trap transgenic lines could be instrumental for developmental studies of CVOs in zebrafish and understanding of the molecular mechanism of disease such a hydrocephalus in human. Their future analysis may shed light on general and specific molecular mechanisms that regulate development of CVOs.

## Introduction

It was noted that “the circumventricular organs (CVO) are peculiar brain structures that are located in the walls and often protrude in the lumen of the third and fourth ventricles (Hofer, 1958)” and that “no clear agreement can be found in the literature on the number of these organs in mammals” (Duvernoy and Risold, 2007). The adult CVOs are highly vascularized, but the development of vascularisation is not studied in detail. Unlike that in the rest of the brain their blood-brain barrier (BBB) is semitransparent. This allows the specialized cells of CVOs to directly sense the chemical composition of blood and secrete hormones into systemic circulation. Thus, the CVOs were nicknamed “windows to the brain” (Weindl and Sofroniew, 1981; Johnson and Gross, 1993). They perform diverse functions, including, but not limited to, the exchange of information between blood, brain and cerebrospinal fluid (CSF), generation of CSF with all its specialized proteins, etc. (Cottrell and Ferguson, 2004; Joly et al., 2007). More recently, it has been found that the CVOs, including the choroid plexus (CP), act as brain-immune interfaces mediating the transfer of immune cells from blood to brain (Shimada and Hasegawa-Ishii, 2017). This is in line with recent attempts to systematize the CVOs while considering microglia to be an important component of these structures (Oldfield and McKinley, 2014; Miyata, 2015; Kaur and Ling, 2017).

The whole range of CVOs' functions remains not fully understood even in model animals. Nevertheless, it includes many functions crucial for life support, as regulation of body fluids, temperature and energy balance, pain, brain detoxification, and so on. In mammals, there are nine CVOs: the pineal gland (PIN) or epiphysis [with the separate parapineal organ (PP) in some species, including zebrafish], subfornical organ (SFO), organum vasculosum laminae terminalis (OVLT), paraventricular organ (PVO), median eminence (ME), neurohypophysis (NH), subcommissural organ (SCO), and area postrema (AP). Some authors also add to this list the CP (Joly et al., 2007; García-Lecea et al., 2008; Wilson et al., 2010). At another extreme an extensive study described “about 17 different CVOs” in 31 species belonging to various groups of vertebrates, from cyclostomes to mammals (Tsuneki, 1986). This analysis suggested that the NH, ME, SCO, and PIN, found in almost all vertebrate species examined, could be the oldest CVOs, whereas the SFO and AP could have appeared in evolution relatively recently. It is thought that some CVOs are specialized to sense the chemical composition of blood. These were defined the “sensory” CVOs (OVLT, SFO, and AP). Several other CVOs coordinate brain responses by secreting hormones into peripheral blood stream. These were defined the “endocrine” or secretory CVOs (PIN, PP, NH, ME, and SCO; Ganong, 2000; Duvernoy and Risold, 2007). Despite the grouping of CVOs as specialized organs or their separation into different functional groups, the molecular mechanisms underlying such classifications remain insufficiently studied. As the first attempt in this direction the transcriptome of PIN, SCO, and SFO was analyzed using laser capture microdis section with an eye on a potential role of these CVOs as sites of periventricular tumors (Szathmari et al., 2013) and transcriptomics of the telencephalic and hindbrain CPs detected their regional specificity (Lun et al., 2015). Hence future studies may provide evidence helpful to characterize and/or classify CVOs.

The developmental analysis of CVOs in terrestrial vertebrates until now has been rather limited for the following reasons: the CVOs are small and inconspicuous, develop relatively late and their analysis is largely based on fixed material. Nevertheless, the development of CVOs has been described in different species, including the PIN (Calvo and Boya, 1981), OVLT (Szabó, 1983), SFO (Castaneyra-Perdomo et al., 1992), NH and ME (Ugrumov et al., 1989; Sasaki et al., 2003), PVO (Vigh and Vigh-Teichmann, 1998), CP (Sturrock, 1979; Dziegielewska et al., 2001), and AP (Borison, 1989; Castaneyra-Perdomo et al., 1992). The CP development was described in mice (Louvi and Wassef, 2000; Awatramani et al., 2003; Landsberg et al., 2005; Hunter and Dymecki, 2007).

The zebrafish is a popular model of developmental studies and, notably, *in vivo* analyses pave the way for addressing development of CVOs. The enhancer-trap transgenics represent useful tools of *in vivo* analysis of developing brain. Several enhancer-trap screens yielded a significant number of transgenic lines expressing various markers, including the cytosolic green fluorescent protein (GFP) (Parinov et al., 2004; Kondrychyn et al., 2009) or membrane-tethered KillerRed (Teh et al., 2010). In the zebrafish, development of some of CVOs has been studied, including the PIN (Masai et al., 1997), PIN and PP (Concha and Wilson, 2001), NH and ME (Bassi et al., 2016), PVO (Xavier et al., 2017), SCO (Fernández-Llebrez et al., 2001), AP (Ma, 1997; Holzschuh et al., 2003), and CP of the IIIrd and IVth ventricle (Bill et al., 2008; García-Lecea et al., 2008; Bill and Korzh, 2014). The study of CP development relied on *in vivo* analysis of the developing brain in the Gateways transgenics (ET33-E20). In this transgenic line, the GFP is also expressed in migratory microglia as well as brain regions other than CP (García-Lecea et al., 2008). Mapping of these unidentified regions based upon published literature and neuroanatomical landmarks suggested that in addition to the CP and migratory microglia the GFP expression domains may represent at least six other CVOs of zebrafish–the OVLT, SFO, AP, ME, PVO, PIN, i.e., most of CVOs of zebrafish, whereas in two other lines GFP expression was detected in the PP and SCO. In parallel, one of the transgenic lines expressing KR-KR19 was shown to express this marker in the CP (Teh et al., 2010; Korzh et al., 2011) and several other regions reminiscent of those in Gateways in indication of a similar developmental regulation of these independent markers. This illustrates the utility of the enhancer trap zebrafish transgenics for the neuroanatomical and developmental analyses of small brain structures. Given the scarcity of genetic information related to development of CVOs and the possibility to perform *in vivo* developmental analysis, these transgenics represent a very useful resource, which potential still remains to be exploited in more detail.

## Materials and methods

### Animals

Zebrafish were maintained according to established protocols (Westerfield, 2007) in agreement with Institutional Animal Care and Use Committee regulations (the Biological Resource Center of the Biopolis, Singapore, license no. 120787) that approved the study and rules of the zebrafish facility at the Institute of Molecular and Cell Biology, Biopolis, Singapore. All experiments in Singapore and Warsaw involving zebrafish embryos/larvae were carried out in accordance with the IACUC rules. The zebrafish transgenics used in this study (*sqet33e20ET* (referred here to as ET33-E20 or Gateways), *sqet33b13, sqet22-1, sqet27, sqet33-10*) express cytosolic GFP, *sqKR19ET* expresses membrane-tethered KillerRed (Parinov et al., 2004; García-Lecea et al., 2008; Kondrychyn et al., 2009; Teh et al., 2010), *Tg(kdrl:ras-cherry)*^*s*916^ expresses Cherry in developing vasculature (Krueger et al., 2011).

### Live imaging

Pigmentation od zebrafish was inhibited with 0.2 mM 1-phenyl-2- thiourea (PTU) in egg water. For imaging, embryos were dechorionated at the selected stages, anesthetized with 0.02% tricaine and oriented by embedding in 0.8% low melting agarose (LMA) in embryo water on a glass coverslip floor of a small petri dish plate. While the agarose was still liquid, embryos were positioned with two needles and left for 5–10 min at room temperature until agarose set and was hard enough to hold the embryo. All embryos held in the imaging chamber maintained heartbeat and circulation throughout the imaging period. Microscopic observations were performed using a dissecting fluorescent microscope SZX12 (Olympus, Japan) and a compound microscope Zeiss Axioscope2.

### Confocal laser scanning microscopy

The temperature of the microscope chamber was maintained at 28°C during image acquisition. Imaging was performed using the microscope Zeiss LSM 800 with Airyscan (Carl Zeiss, Germany). 488 and 561 nm lasers were used to excite fluorescence with emission detected using emission filters (505–545 and 575–615 nm BP), respectively. Data were saved in the CZI format and then processed using ImageJ 1.51n software (Fiji). For each z-stack average intensity and sum slices projections were generated.

### Light-sheet fluorescence microscopy imaging

Embryos and larvae were anesthetized and embedded into a glass capillary with a plunger (~1 mm inner diameter, Zeiss) filled with 1% low-melting point agarose in E3 medium. Once agarose fully polymerized, capillary was mounted in sample holder and placed in microscope chamber filled with E3 0.02% tricaine and then short part of the agarose column containing a specimen was pulled out. The temperature of the microscope chamber was maintained at 28°C during image acquisition. Imaging was performed using the microscope ZEISS Lightsheet Z. 1 with W Plan-Apochromat 20x/1.0 UV-VIS objective. 488 nm and 561 nm lasers were used to excite fluorescence with emission detected using 505–545 and 575–615 nm BP emission filters, respectively. Data were saved in the LSM format and then processed using ZEN software (Zeiss). For each z-stack maximum intensity projections were generated.

### Whole mount *in Situ* hybridization (WISH), fluorescent immunohistochemistry and histology

Embryos were processed for WISH, cryo-sectioning, and immunohistochemistry as before (Korzh et al., 1998). The following antibodies were used: mouse monoclonal anti-GFP antibodies (clone B-2, Santa Cruz Biotechnology, 1 mg/ml), anti-acetylated tubulin (Sigma–Aldrich, T6793, 2 mg/ml), AFRUMA antibodies (1:500; kindly provided by Prof. J. M. Grondona; (Rodríguez et al., 1984; López-Avalos et al., 1997) and goat anti-mouse/Alexa Fluo488 (Molecular Probes, 2 mg/ml).

### Preparation of schematics

All schematics were drawn using the Adobe Photoshop and Inkscape (version 0.92.2) software.

## Results

### Gateways transgenics express GFP in several periventricular midline regions

SqET33-E20 (Gateways) is the enhancer-trap (ET) line generated by remobilizing the Tol2 transposon-based ET cassette in SqET33 (chr. 14) transgenic line, one of the first-generation enhancer-trap transgenics derived from random insertion of Tol2-based enhancer-trap cassette (Parinov et al., 2004; Kondrychyn et al., 2009). The Gateways line was used to describe development of CP (García-Lecea et al., 2008). A single insertion of the transposon in Chr. 24 detected in the Gateways results in a characteristic expression pattern that remains unassigned to any of the genes found in the vicinity of the insertion (see Table 1 and Discussion). In the developing brain of Gateways embryos GFP is expressed in several periventricular midline regions other than CP, i.e., similar to the roof plate and CP. One feature these regions have in common is the appearance of GFP expression prior to the penetration of capillaries. These regions were found in the ventral forebrain, epithalamus, CP (Bill et al., 2008; García-Lecea et al., 2008), etc. To define GFP expression domains other than CP we began by reviewing their neuroanatomical localization.

**Table 1:** Zebrafish transgenics with expression of fluorescent markers in circumventricular organs.

**Transgenic line**	**insertion site**	**Expression in CVO**	**References**
ET22-1	4.4 kb upstream of *tsr2*, Chr. 8	PIN, PP, MHB	Parinov et al., 2004
ET27	*pard3aa*, intron, Chr.24	SCO	Parinov et al., 2004
ET33-B13	*lrp1ab*, intron, Chr. 23	AP, astroglia CPIV	Kondrychyn et al., 2009
ET33-10	*nocta*, intron, Chr. 14	SCO, CPIII-IV, RP, FP	Kondrychyn et al., 2009, 2013
ET33-E20 (Gateways)	4.2 kb upstream of *csrnp1b*, Chr. 24	CPIII-IV (astroglia and epithelial cells), AP, ME, OVLT, PIN, SFO, RP, MHB	García-Lecea et al., 2008; Kondrychyn et al., 2009
KR19	32,151 bp downstream of *foxp3b*, Chr.8	CPIII-IV, RP, AP, PIN	Teh et al., 2010; Korzh et al., 2011

### Some of GFP expression domains in gateways embryos may represent the “sensory” CVOs

**Figure 1:**
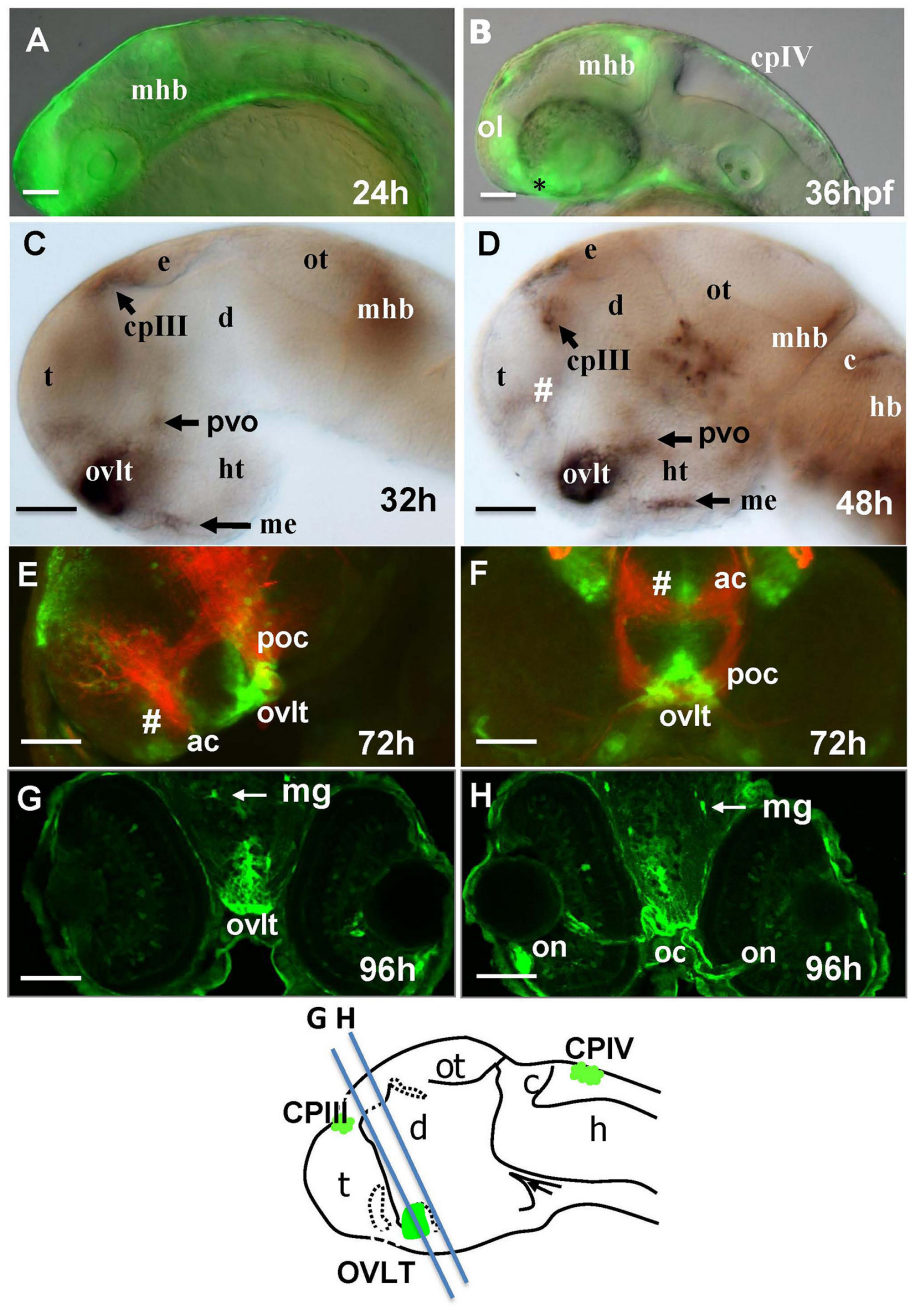
Transgenic zebrafish (Gateways) expresses GFP in several CVOs and migratory microglia. **(A–F)** whole mounts (anterior to the left); **(A,B)** *in vivo*; **(C,D)** anti-GFP WISH (eyes and olfactory placode removed); **(A–E)** lateral view; **(F)** frontal view; **(E,F)** double immunohistochemistry (anti-GFP–green, anti-acetyl-tubulin–red; **(E)** eyes and olfactory placode removed), **(G,H)** anti-GFP immunohistochemistry on cross-sections. Scheme indicates the level of cross-sections shown in **(G,H)**. ac, anterior commissure; ah, adenohypophysis; c, cerebellum; cpIII, choroid plexus of III ventricle; cpIV, choroid plexus of IV ventricle; d, diencephalon; e, epiphysis; h, hour postfertilization; hb, hindbrain; ht, hypothalamus; mhb, midbrain-hindbrain boundary; me, median eminence; mg, migratory microglia; oc, optic chiasm; ol, olfactory placode; on, optic nerve; ot, optic tectum; ovlt, organum vasculosum laminae terminalis; poc, postoptic commissure; t, telencephalon; tc, tela choroidea; #, subfornical organ. Scale bar−50 μm.

**Figure 2:**
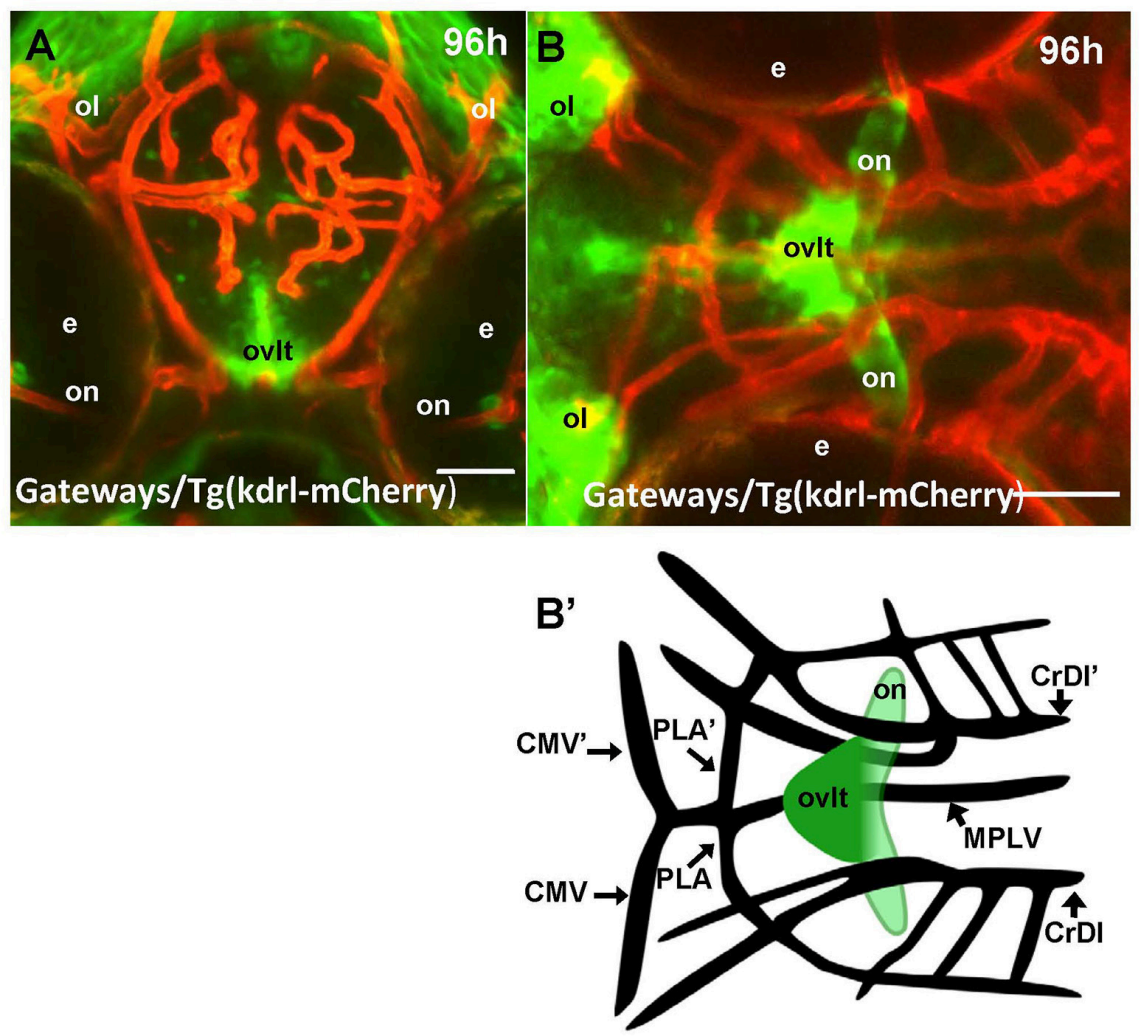
Four days old Gateways larvae express GFP in the OVLT. Confocal microscopy of compound transgenics Gateways (GFP, green)/Tg (kdrl:ras-Cherry) (red) *in vivo* reveals the OVLT in respect of developing vasculature. **(A)** Frontal view at the level immediately anterior to the optic chiasm; **(B)** dorsal view at the level of OVLT; **(B')** schema of the OVLT in respect of vasculature (based on the vasculature atlas, Isogai et al., 2001). cmv, communicating vessel; crdi, cranial division of the internal carotid artery; d, diencephalon; ht, hypothalamus; e, eye; mplv, median palatocerebral vein; oc, optic chiasm; ol, olfactory placode; on, optic nerve; ovlt, organum vasculosum laminae terminalis; pla, palatocerebral artery. Scale bar−50 μm.

One of the most prominent sites of GFP expression is in the ventral forebrain of Gateways (Figures 1A,B). At 24–36 hpf, the detailed analysis of the ventral forebrain is difficult on whole mounts *in vivo*, since this area is blocked by surrounding tissues. To observe this region without obstruction, we analyzed the distribution of *gfp* mRNA using whole mount *in situ* hybridisation (WISH) at 32 and 48 hpf after microsurgically removing surrounding tissues (Figures 1C,D). This helped to reveal and correctly map the strong signal found anterior to the optic chiasm. Interestingly, in this position the OVLT has been mapped in adult zebrafish as one of the semitransparent BBB sites (Jeong et al., 2008). To define this area in more detail, 72 hpf larvae were double-stained with anti-GFP (green)/anti-acetyl-tubulin (red) antibodies, which revealed two midline signals, one dorsal and anterior to the anterior commissure (# in Figures 1E,F) and another one located ventrally in the preoptic area with its anterior limit posterior to the anterior commissure, and its posterior edge limited by the postoptic commissure (Figures 1E,F). 96 hpf larvae were cryo-sectioned and GFP expression detected by anti-GFP antibodies (Figures 1G,H). This confirmed the localization of the second (posterior) domain of GFP-positive cells to the ventral midline of the preoptic area anterior to the optic chiasm (Figures 1G,H, 2A,B). Based on their neuroanatomical position these two signals may represent the SFO (anterior signal) and OVLT (posterior signal). These CVOs are known as “sensory” CVOs with similar neuroanatomical organization. It was noted that these two organs share location at the laminae terminalis being separated by the anterior commissure (Duvernoy and Risold, 2007) with the SFO found in more anterior and dorsal position. WISH and live imaging detected a weak signal in this location from 48 hpf onwards (Figure 1D, Figure S1A). The whole mount two-color immunohistochemistry of Gateways transgenics clearly revealed a small group of GFP-positive midline cells anterior to the anterior commissure (Figures 1E,F), i.e., neuroanatomical location corresponding to that of the SFO. At this stage, these two domains are separated. The ventral domain is closely associated with cranial vasculature (Isogai et al., 2001; Figures 2A,B).

Mutant analysis is commonly used to tackle the developmental mechanism behind development of various cell lineages and organs. The zebrafish mutants *ace*^−/−^ are deficient in FGF8a (Fürthauer et al., 1997), *mib*^−/−^ in Notch (Itoh et al., 2003), *mbl*^−/−^ in Wnt (Heisenberg et al., 2001) and *smu*^−/−^ in Hh signaling (Chen et al., 2001). At 48 hpf both expression domains were missing in *ace* (Figures S1C,D) and *smu* (Figures S1I,J) mutants demonstrating a requirement in FGF8a and Hh for development of these domains. In particular, the Hh signaling has been shown earlier to be essential for development of several cells types at the ventral midline such as the lateral floor plate, motoneurons, etc., (Roelink et al., 1994; Strähle et al., 2004). Similar to Hh, Fgf8 seems to be involved in the patterning during brain development (Sleptsova-Friedrich et al., 2001; Wang et al., 2003). Compared to the ventral domain, the dorsal one was more significantly affected in *mib* (Figures S1E,F) and *mbl* (Figures S1G,H), pointing to a different role of the Wnt and Notch signaling in development of these two regions. This could be linked to the fact that the ventral domain seems to be larger and develop earlier compared to dorsal one. Therefore, it could be less vulnerable to depletion of early progenitors and patterning defects taking place in *mib* mutants (Itoh et al., 2003; García-Lecea et al., 2008).

At 72 hpf the signal detected by WISH is more intense in the ventral domain (Figures S2A,A'). This staining also revealed that the ventral domain consists of several clusters of cells with variable expression of *gfp* mRNA (Figure S2A', ^*^). By 144 hpf, the two domains appear as a single GFP-positive cluster in the preoptic area in the shape of seahorse (Figure S2B, Figure 8, Supplementary Movie 1), which anterior and dorsal part, i.e., the “seahorse” head is significantly reduced in the Notch-deficient *mib* mutants similar to that at early stages (Figures S1E,F), whereas the ventral and posterior part (“seahorse” trunk) despite being somewhat deformed is less affected (Supplementary Movie 2).

**Figure 3:**
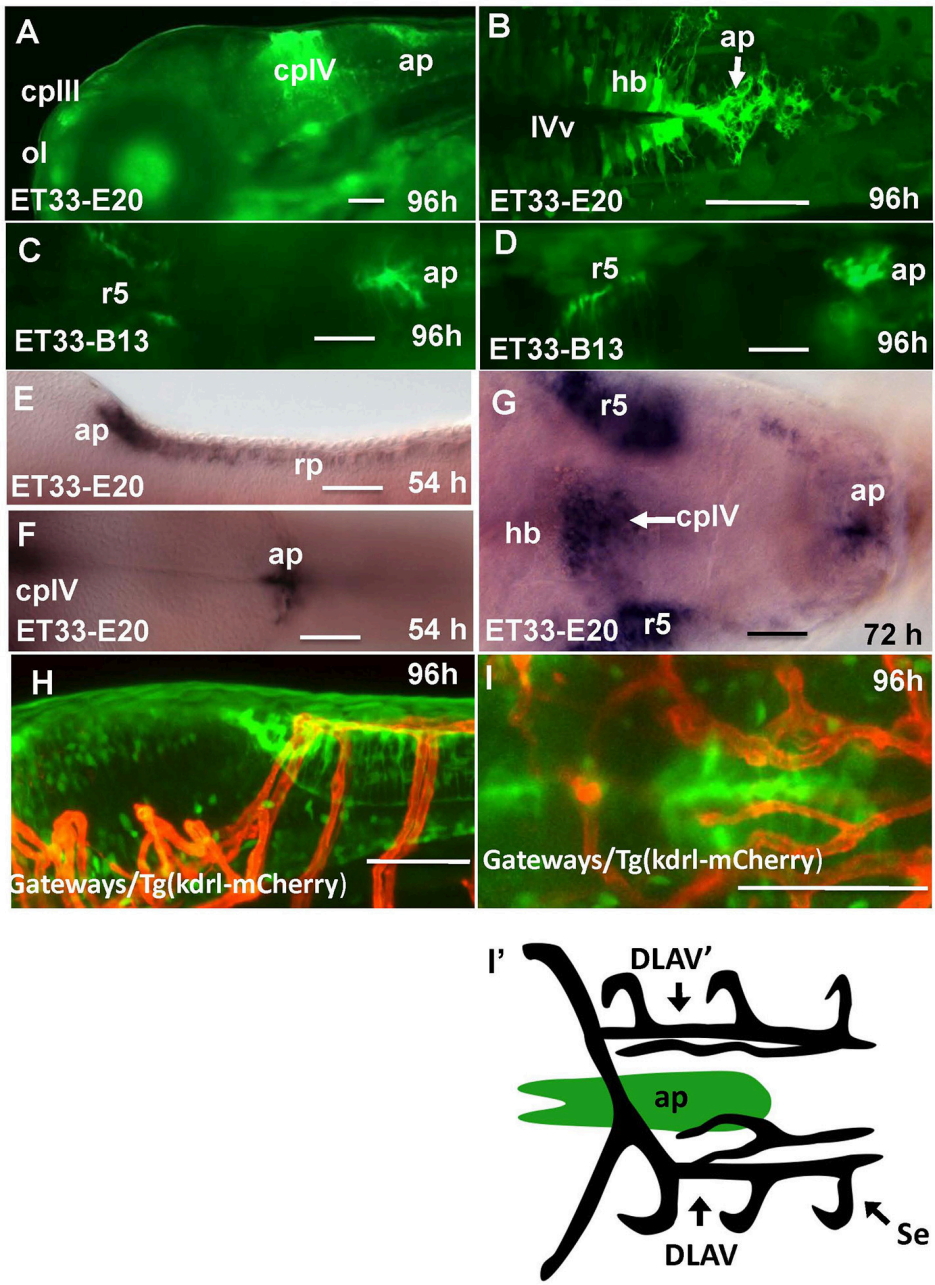
Transgenic zebrafish lines express GFP in the AP as detected by GFP *in vivo*. Being a derivative of the roof plate, the AP is located at the dorsal hindbrain-spinal cord junction. In the Gateways transgenics the GFP expression domain in this area is rather broad unlike that in the ET33-B13 transgenics. All images (except **I'**, which is a scheme based on **I**) are of whole mounts (anterior to the left). **(A,D,E,H)** lateral view, **(B,C,F,G,I,I')** dorsal view. **(A–D,H,I)** *in vivo* whole mounts. ap, area postrema; cpIII, choroid plexus of III ventricle; cpIV, choroid plexus of IV ventricle; dlav, Dorsal longitudinal anastomotic vessel; h, hour postfertilization; hb, hindbrain; ol, olfactory placode; ot, optic tectum; r5, rhombomere 5; se, intersegmental vessel; IVv, IV ventricle. Scale bar−50 μm.

In the hindbrain of 54–96 hpf Gateways larvae two major clusters of GFP domains of expression were detected (Figures 3A,E–G). The larger anterior signal (Figures 3A,G) corresponds to the CPIV (García-Lecea et al., 2008). The small posterior domain is located at the junction of the caudal hindbrain and anterior spinal cord (Figures 3A,B,E,F). The roof plate cells in the posterior hindbrain are oriented along the mediolateral axis, and in the anterior spinal cord they change their orientation along the dorsoventral axis. At the hindbrain-spinal cord junction such cells form the characteristic “bouquet” (Figures 3C,D). The ET33-B13 transgenics express GFP in the lateral clusters of CPIV plus at the caudal hindbrain-anterior spinal cord junction (Figures 3C,D) albeit in a much more restricted fashion. This location is known to contain the AP (Ma, 1997; Holzschuh et al., 2003). Similar to the GFP expression domain in the pre optic area, the domain at the hindbrain-spinal cord junction is closely associated with dense network of developing vasculature (Isogai et al., 2001; Figures 3H,I,I'). Taken together, this analysis suggested that Gateways may express GFP in all three “sensory” CVOs of zebrafish-OVLT, SFO and AP.

### Gateways embryos may express GFP in some “endocrine” CVOs

**Figure 4:**
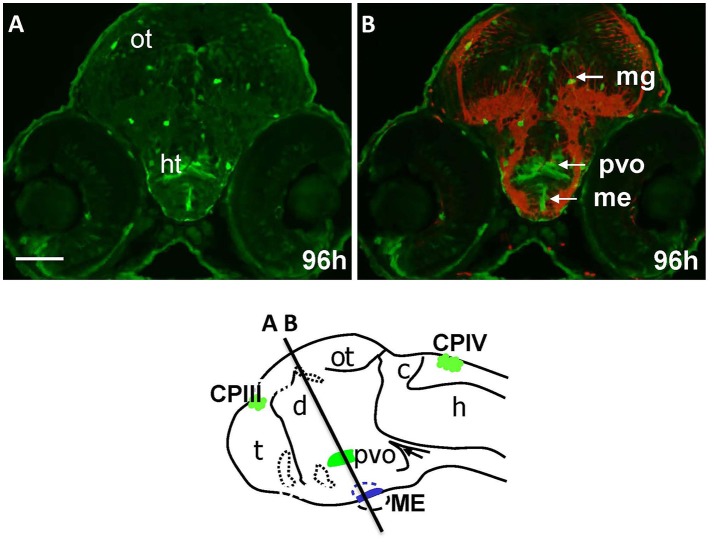
Gateways express GFP in the developing hypothalamus. Cross-sections of Gateways larvae stained by immunohistochemistry. The ME is in the ventral midline position in contrast to the PVO, which is more dorsal and in lateral walls of hypothalamus. Scheme indicates the level of cross-sections shown in **(A,B)**. **(A,B)** Cross-sections, **(A)** anti-GFP; **(B)** anti-GFP (green)/anti-acetyl-tubulin (red). Scale bar−50 μm.

The “endocrine” CVOs consist of the NH, ME, PIN (epiphysis), PP, SCO and PVO. WISH in Gateways revealed the ventral midline signal in the hypothalamus. This signal, elongated along the anterior-posterior axis, is detected by WISH in a single cell layer at 32 and 48 hpf, which maps immediately above the adenohypophysis (Figures 1C,D). *gfp* expression at this site becomes less obvious at 72 hpf (Figure S2A). The same ventral midline domain in the hypothalamus could be seen at 96 hpf cross-sections stained for immunohistochemistry (Figures 4A,B). Based on its location between the adenohypophysis and hypothalamus (Bassi et al., 2016), this domain may correspond to ME. The same section (Figures 4A,B) demonstrated GFP expression in solitary cells representing migratory glia described previously in Gateways (García-Lecea et al., 2008) and another GFP-positive area represented by the bilateral domain in the hypothalamus. Such neuroanatomical position is consistent with description of the PVO (Figure 4B). *gfp* expression in this site has been detected by WISH (72 hpf, Figure S2A).

**Figure 5:**
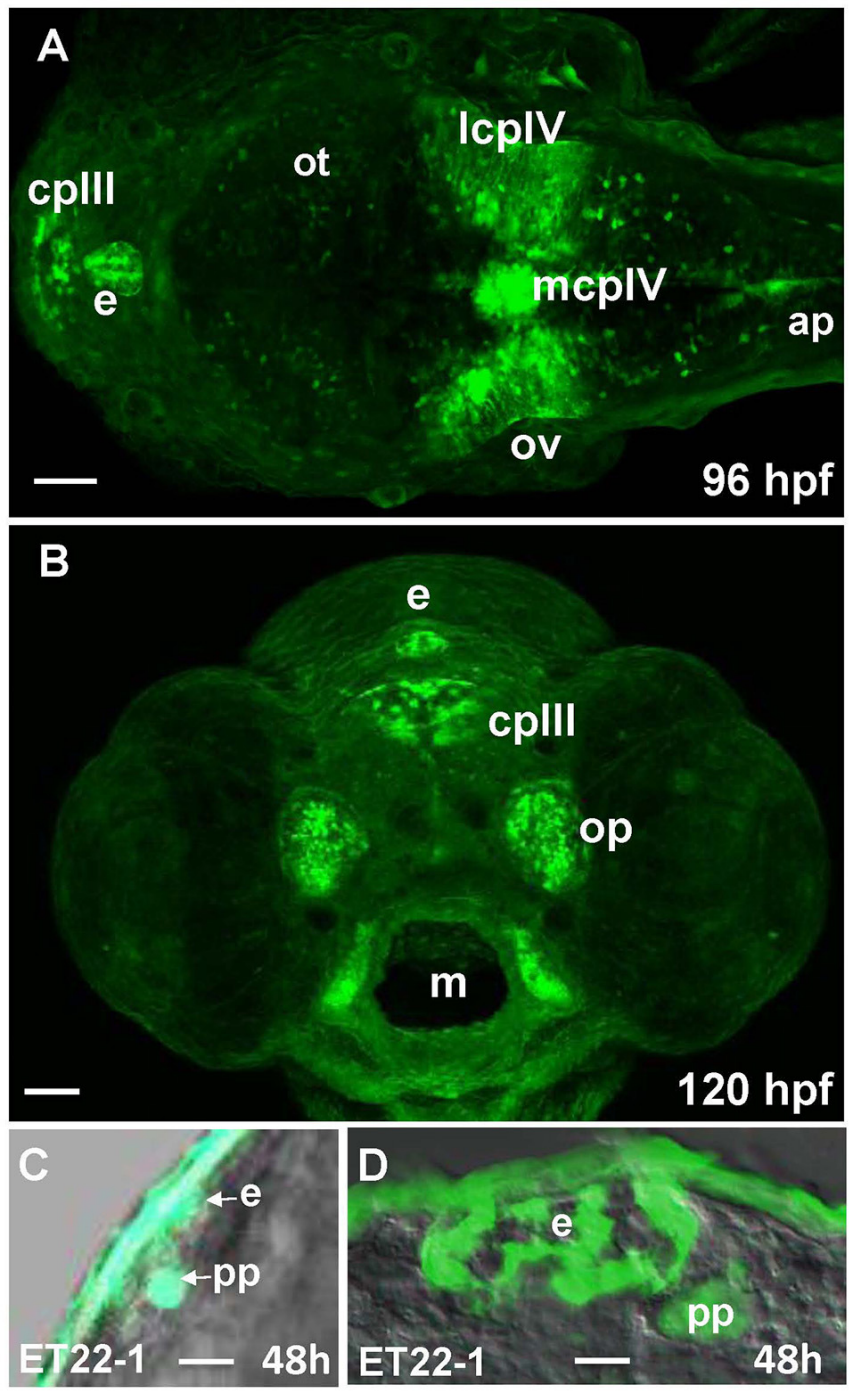
Gateways and ET22-1 transgenics express GFP in the developing epiphysis (pineal). **(A,B)** Gateways; **(C,D)** ET22-1. **(A–C)** whole mounts, **(D)** cross-section. **(A)** Dorsal view; **(B)** frontal view (superficial scan); **(C)** lateral view; **(A–C)** *in vivo*, **(C)** anti-GFP immunohistochemistry. e, epiphysis; h, hour postfertilization; lcpIV, lateral cluster choroid plexus of IV ventricle; m, mouth; mcpIV, medial cluster, choroid plexus of IV ventricle; op, olfactory pit; ov, otic vesicle; pp, parapineal. Scale bar−50 μm **(A,B)**, 25 μm **(C)**, 10 μm **(D)**.

96 hpf Gateways larva expresses GFP in the PIN (epiphysis; Figures 5A,B). This expression is similar to that in the ET22-1 (Figures 5C,D). An observation of the ET22-1 forebrain in lateral projection reveals expression not only in the epiphysis, but in the PP also (Figure 5C) as confirmed by immunohistochemistry on sections (Figure 5D). The pineal (epiphysis)-parapineal complex exists in some species (e.g., in humans) as a single organ. Nevertheless, in zebrafish the two glands have been detected. The epiphysis is a well-known regulator of circadian rhythms, whereas the parapineal gland regulates the left-right asymmetry of the brain (Cau, 2003; Concha et al., 2003; Gamse, 2003; Halpern et al., 2003).

**Figure 6:**
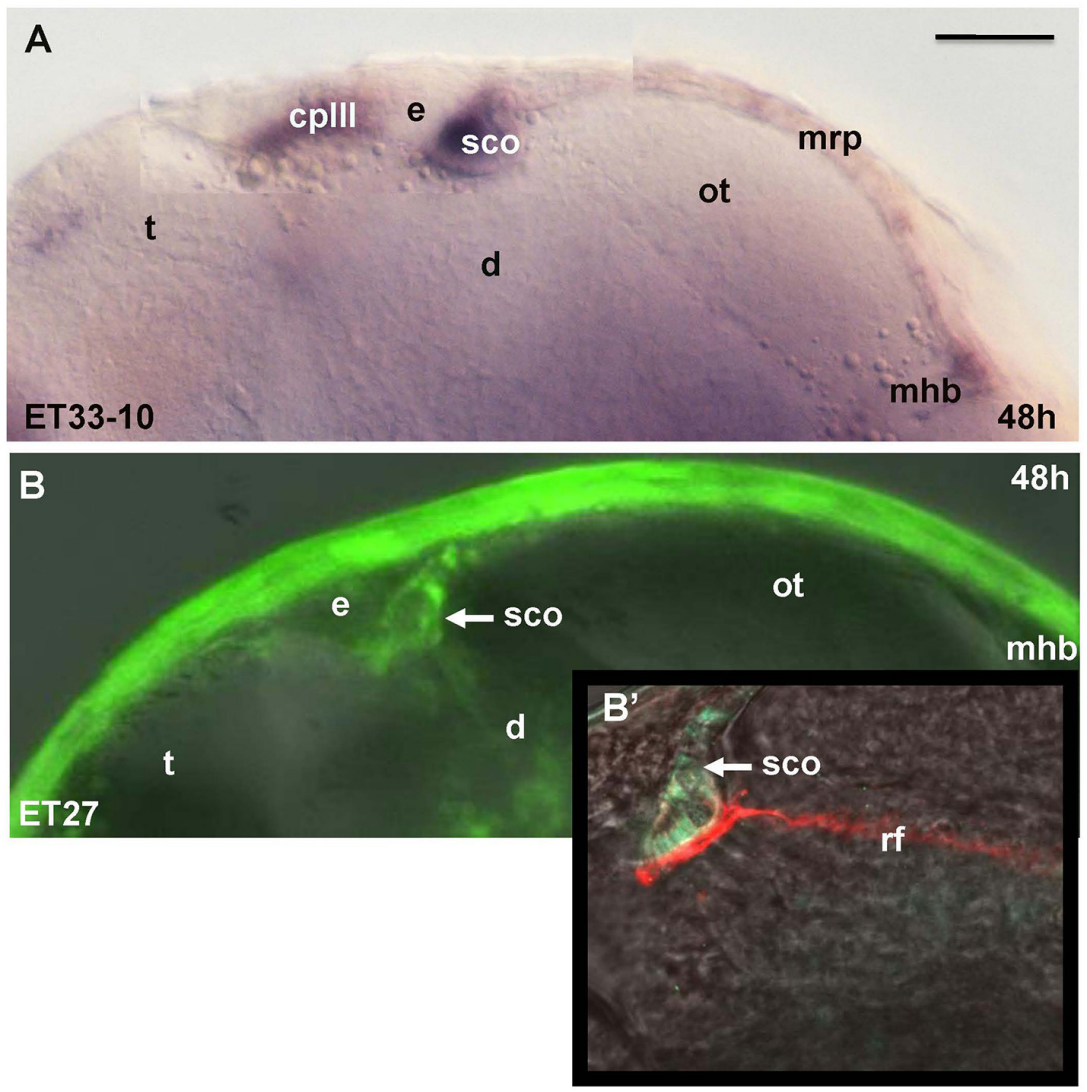
Transgenic zebrafish lines (ET33-10, ET27) express GFP in the SCO, which represents the anterior bulging of the midbrain roof plate. The SCO expression detected by WISH (magenta, **A**) or by confocal microscopy *in vivo* (green-**B,B'**). Sco-spondin forms the Reissner fiber detected by anti-Reissner fiber antibody (red, **B'**). All images are of whole mounts in lateral view (anterior to the left). **(A)** WISH; **(B)** confocal microscopy *in vivo*; **(B')** two-color immunohistochemistry; **(A)** ET33-10; **(B,B')** ET27. cpIII, choroid plexus (IIIrd ventricle); d, diencephalon; e, epiphysis (pineal); h, hour postfertilization; mhb, midbrain-hindbrain boundary; mrp, midbrain roof plate; ot, optic tectum; rf, Reissner fiber; sco, subcommissural organ; t, telencephalon. Scale bar−50 μm.

**Figure 7:**
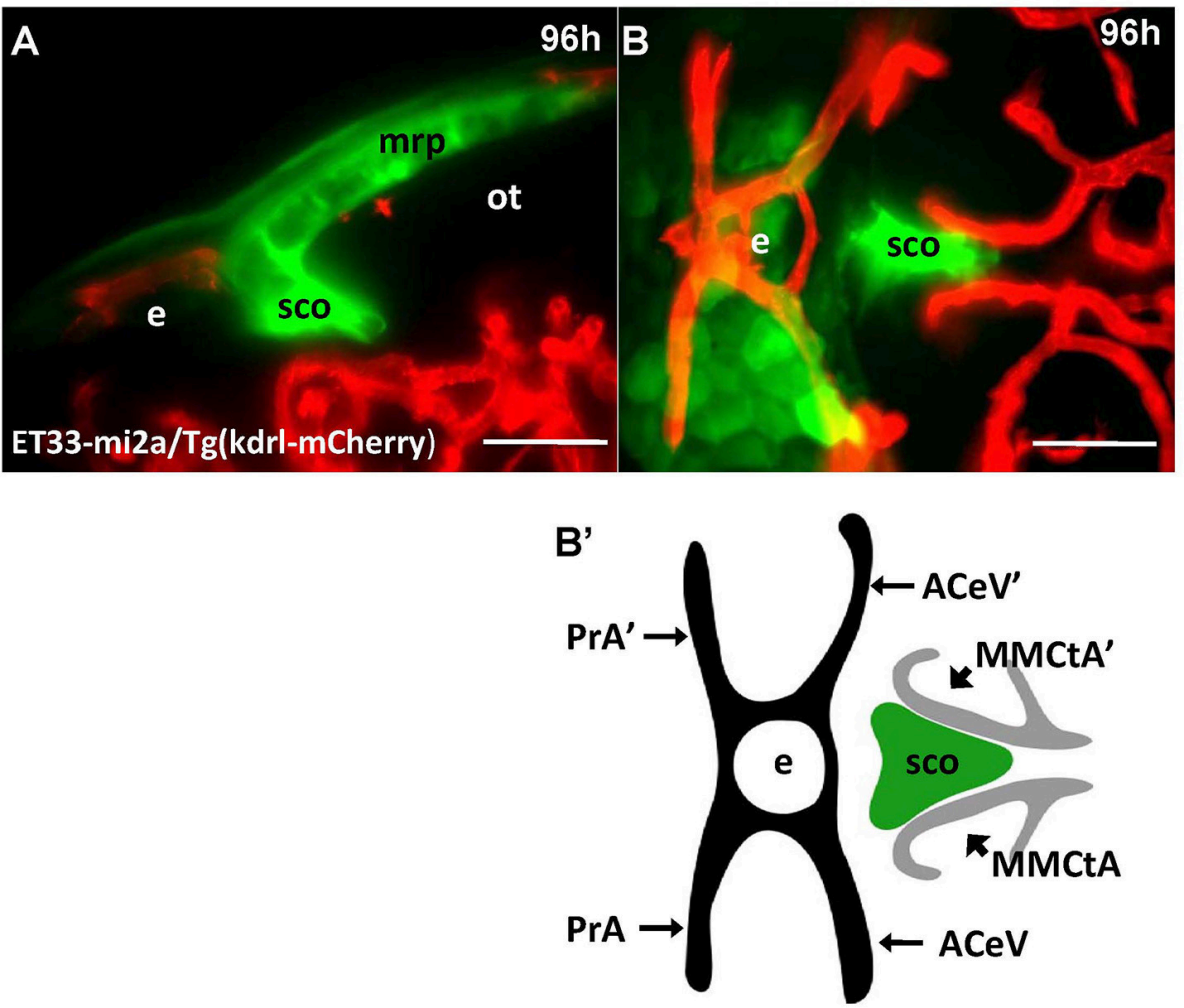
Four days old ET33-mi2A zebrafish larva express GFP in the SVO. Confocal light sheet microscopy of compound transgenics ET33-mi2A (GFP, green)/Tg (kdrl:ras-Cherry) (red) *in vivo* reveals the SCO in respect of developing vasculature. **(A)** lateral midline view; **(B)** dorsal view; **(B')** schema of the relative position of the SCO in respect of vasculature (based on the vasculature atlas, Isogai et al., 2001). acev, anterior (rostral) cerebral vein; d, diencephalon; e, epiphysis; mmcta, middle mesencephalic central artery; mrp, midbrain roof plate; ot, optic tectum; pra, prosencephalic artery; sco, subcommissural organ. Scale bar−50 μm.

Unlike Gateways several enhancer-trap transgenic lines (ET33-10; ET27) express GFP in the anterior extent of midbrain roof plate posterior to the epiphysis and ventral to the posterior commissure (Figure 6). In the 48 hpf ET33-10 embryos this signal could be mapped using WISH (Figure 6A), whereas in the 48 hpf ET27 embryos by confocal *in vivo* imaging and two-color immunohistochemistry (GFP-AFRUMA; Figures 6B,B'). This neuroanatomical position corresponds to the SCO and this region is specifically detected by the anti-Reissner fiber antibody (Fernández-Llebrez et al., 2001) to prove that this region represents the SCO of embryonic zebrafish. Similar to GFP domains in the pre-optic area and hindbrain-spinal cord junction closely associated with developing vascular network, which contain the OVLT and AP, correspondingly, the epiphysis and SCO both are closely surrounded by developing capillaries (Isogai et al., 2001; Figures 7A,B,B').

The anatomical and functional information on CVOs obtained after the detailed analysis of GFP expression in Gateways and its mutants, set ground to review the identity of some domains of expression of KR in KR19 and KR19/Gateways crosses. The combined expression pattern showed that besides of the CPIII and IV, KR is expressed at least in the PIN, AP and the spinal cord roof plate (Figure S3), whereas more study is required to explore this expression pattern in detail. Given the properties of KR as the optogenetically-inducible donor of reactive oxygen species (Lee et al., 2010; Teh et al., 2010), this new zebrafish cross opens the possibility to manipulate development of at least some, if not most of, CVOs by optogenetically induced oxidative stress.

**Figure 8:**
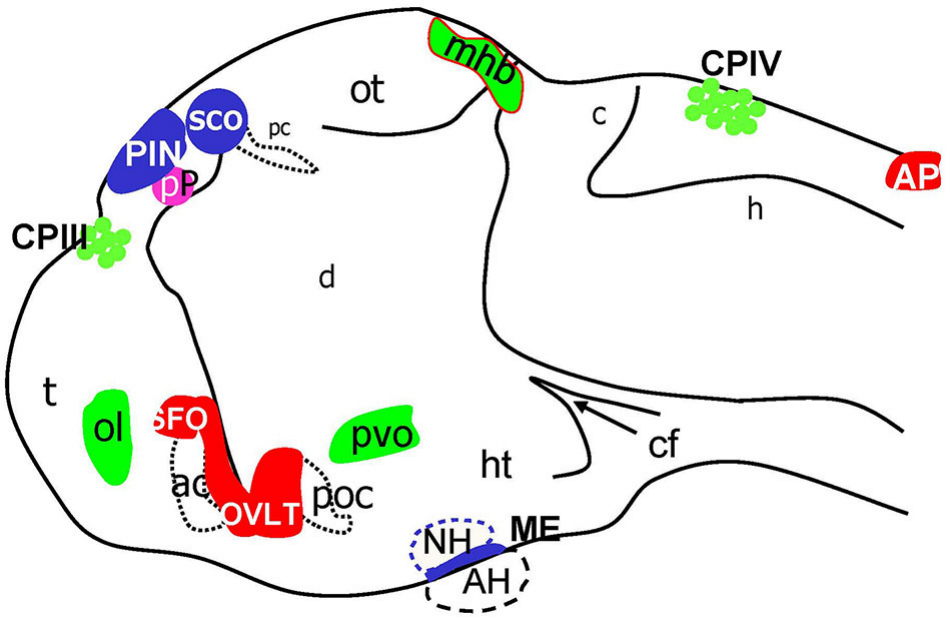
Schematic of CVOs detected using ET transgenics in 4 days old zebrafish brain (96 hpf). ac, anterior commissure; ah, adenohypophysis; ap, area postrema; asterisk, ovlt (organum vasculosum of the lamina terminalis); c, cerebellum; cf, cephalic flexure; cpIII, choroid plexus of III ventricle; cpIV, choroid plexus of IV ventricle; d, diencephalon; h, hindbrain; ht, hypothalamus; mhb, midbrain-hindbrain boundary; oc, optic chiasm; ot, optic tectum; pc, posterior commissure; pin, pineal gland; poc, post optic commissure; pp, parapineal gland; r5, rhombomere 5; sco, subcommissural organ; t, telencephalon.

Taken together the results of this analysis of Gateways and other transgenics expressing GFP during development allowed to map several GFP expression domains to positions, which correspond to the CP, OVLT, SFO, AP, ME, SCO, PIN, and Pp (Figure 8). Importantly, prior to formation of CVO some of these lines (Gateways, ET33-10) also express GFP in the longitudinal morphogenetic centers of the neural tube-the roof plate and floor plate.

## Discussion

The embryos and larvae of Gateways express GFP in all three “sensory” CVOs-OVLT, SFO and AP, and “specialized” CVO-CP as well as in some, but not all, “endocrine” CVOs-ME, PVO, and PIN. It also expresses GFP in some regions that are not considered to be CVOs-the main longitudinal signaling center of the dorsal neural tube-roof plate, the regional signaling center–MHB, olfactory pits, optic nerve and migratory microglia. This establishes the “Gateways”-regulatory transcriptional machinery as the developmental integrator of most of CVOs. Interestingly, GFP expression in Gateways embraces CVOs of different subgroups and different cell lineages. In the CP GFP expression encompasses at least a couple of cell types, i.e., epithelial cells of the medial cluster and astrocyte-like cells of the lateral clusters (García-Lecea et al., 2008; Figure 5A), but not the vasculature. The fact that there is similar developmental regulation acting upon the AP, PIN, ME, OVLT, SFO, PVO, on one hand, and CP, on the other hand, further supports the notion that CP should be considered as one of CVOs, perhaps, the most divergent one. Intriguingly, while analysis of GFP expression in Gateways at the stages studied suggests some similarity of the CP and other CVOs, it, for reasons currently unknown, segregates the GFP-negative SCO and NH from the rest of CVOs. Perhaps, it indicates that the classification of CVOs must be more complex.

The expression of Gateways transgene seems to reveal developmental events common to the CVOs and roof plate, i.e., it may reflect a more general regulation compared to those events behind the separation of CVOs and roof plate. Thus, an enhancer (still to be cloned and sequenced) may play a role of a transcriptional node upon which expression of genes active in a context of CVO of different functional classes may rely upon. None of several hundreds of transgenic lines analyzed except KR19, which to a large extent phenocopies the expression pattern of Gateways (Teh et al., 2010; Korzh et al., 2011; Yan et al., 2012) show such broad expression in CVOs (Kondrychyn et al., 2009). The Gateways “enhancer” seems to be active early or very sensitive or both since in the CPIV it reveals the very early events of morphogenesis of the CP primordium-*tela choroidea*. If this idea is correct, this enhancer could be the early acting one that regulates the roof plate, MHB, most of CVOs, olfactory pits and migratory microglia. To be validated this idea should be studied further by subjecting Gateways along with KR19 to more detailed analysis aiming to isolate genomic regions regulating development of CVOs and study their role during development and disease of these structures.

The CVOs of adults are highly vascularized (Duvernoy and Risold, 2007). In contrast, the embryonic CVOs remain avascular in zebrafish development despite capillaries pass close by the GFP areas. The *tela choroidea* connects to a capillary, but it is not penetrates inside this area well before the morphologically recognized CP is formed (García-Lecea et al., 2008). Notably, early domains of GFP expression in Gateways look like preferred areas for capillaries to penetrate, whereas our detailed analysis demonstrated that during embryogenesis and early postnatal development vasculature develops in close proximity, but outside of GFP expression domains, which remain avascular (Figures 2, 3, 7). It could be due to various reasons, which may include, but not be limited to, a requirement for CVO to differentiate relying largely on local cues. Further to that, being small zebrafish embryos rely on oxygen diffusion and survive without circulation for several days. Hence the developing CVOs being even smaller may develop due to diffusion from closely located capillaries.

The CVOs share their origin with cells of signaling glia, which constitute the dorsal axial morphogenetic center of the neural tube-the roof plate (Chizhikov and Millen, 2005; Korzh, 2007). In this context it is of interest that the CVOs also is a source of periventricular tumors (Szathmari et al., 2013). In view of scarcity of information about genes regulating CVO development, it might be useful to consider as candidates genes near to the transgene insertions site in transgenic lines analyzed. These genes could be expressed in the CVOs and play a role in their development (Table 1). For example, the analysis of genes found in the vicinity of the Gateways transgene insertion on Chr. 24 (García-Lecea et al., 2008; Kondrychyn et al., 2009) brought into limelight *sulf1*. In parallel, this gene was found to be expressed in the CP and elsewhere in the brain and plays a role in establishing the VegfA-mediated arterial venous identity (Gorsi et al., 2010, 2014). Importantly, the *sulf1* loss-of-function causes deficiency of the IVth brain ventricle (Gorsi et al., 2014), which could be attributed to deficiency of the CPIV. This opens a possibility that *sulf1* could be one of the genes regulated by the activity of the same enhancer that drives GFP expression in Gateways. Indeed, the roof plate-derived chondroitin sulfate may be involved in establishing developmental patterning in the neural tube (Butler and Dodd, 2003). Henceforth, *sulf1* could play an early role in the developmental program of the roof plate and at least some CVOs. The expression pattern similar to that of the Gateways was observed in the transgenic line KR19 expressing membrane-tethered KillerRed fluorescent protein, which unlike Gateways (Chr.24) has been mapped to Chr.8 (Teh et al., 2010; Korzh et al., 2011; Yan et al., 2012).

In this study, the Gateways transgenic line was used to analyze development of several CVOs, including the OVLT, SFO, AP, PIN, PVO, and ME. Several CVOs (PIN, PP, CP, SCO, AP) studied previously are localized in the dorsal neural tube, which makes their microscopic observation more convenient. This was a useful feature in looking for similarity in expression pattern of Gateways and KR19. Our analysis of transgenics demonstrates that the AP and SCO represent rather obvious thickenings of the roof plate at the brain boundaries (Figures 3, 6, Figure S2) and could be correctly mapped based both on available neuroanatomical landmarks, specific antibodies (SCO) and prior observations (Ma, 1997; Fernández-Llebrez et al., 2001; Holzschuh et al., 2003). Holzschuh et al. (2003) Mapping of other CVOs-OVLT, SFO, PVO, ME–found in more ventral position is more complicated due to their small size (ME), opacity of surrounding tissues (OVLT) or both (SFO) and is more reliable using more complex combination of microscopic techniques. The adult rat OVLT is located anterior to the IIIrd ventricle (Prager-Khoutorsky and Bourque, 2015), whereas in the developing zebrafish it is localized initially posterior to the ventral-most extent of the IIIrd ventricle, where it forms several closely connected clusters. It looks like while formed as individual cell groups, the OVLT and SFO in later development fuse to form one complex similar to that of the PIN-PP complex (Figure S2). Of interest are different signaling requirements regulating development of these two CVOs. The OVLT being a larger of the two seems to be born earlier and spared of the deleterious effect that the Wnt and Notch deficiencies have on the SFO (Figure S1). Being sensitive to Notch is consistent with the idea that the SFO is formed later that OVLT, when a pool of neural progenitors is depleted (Itoh et al., 2003; García-Lecea et al., 2008). In contrast, the Hh and FGF8a signaling are critical for development of both CVOs (Figures S1C,D,I,J). Such developmental time table is also clear from morphological analysis.

The AP and SFO are considered to be the evolutionarily “young” CVO (Tsuneki, 1986). It is rather gratifying that the SFO, although is rather minute initially, could be identified since it expresses the same transgene as two other “sensory” CVO-AP and OVLT. Unlike the closely related OVLT, until now SFO remained unidentified even in adult zebrafish. This could be due to a fusion with the larger OVLT during the period from 3 to 6 days postfertilisation. Later analysis of genes known to be expressed in the SFO of larger animals (Hindmarch and Ferguson, 2015) may link their expression to the site where the SFO is found in the brain of developing Gateways embryos/larvae.

Despite the ME being found in a very ventral position, counterintuitively it may share its origin with the dorsal midline cells due to the cephalic flexure, which brings cells found during neural plate stage in the anterior and dorsal position into the ventral forebrain later on. In the *Xenopus* much of the ventral forebrain derives from the anterior neural ridge (Eagleson and Harris, 1990). Similarly, in zebrafish the adenohypophysis, which the ME is adjacent to, is of the anterior placode origin and derives from the most anterior tip of the neural plate (Glasgow et al., 1997; Wang et al., 2001). ME progenitors may derive from a site very close to that of the adenohypophysis. Same could be said about the origin of the OVLT/SFO found anteriorly to the ME and adenohypophysis. This means that the OVLT/SFO progenitors during neural plate stage may reside somewhat posterior to the more anterior progenitors contributing to the adenohypophysis and ME. This hypothesis relies solely on the observation of developmental changes in the expression of Gateways transgene. Its experimental validation is beyond the scope of this descriptive study and should wait further experiments.

## Author contributions

Conceived and designed the experiments: MG-L, IK, CT, and VK. Performed the experiments: MG-L, IK, CT, EG, JJ, and M-SY. Analyzed the data: MG-L, IK, CT, EG, JJ, and VK. Wrote and approved the paper: VK.

### Conflict of interest statement

The authors declare that the research was conducted in the absence of any commercial or financial relationships that could be construed as a potential conflict of interest.

## Supplementary material

Figure S1 Mutant analysis of development of the OVLT and SFO at 48 hpf. **(A,B)** Frontal view and confocal cross-section of control embryo. Strong GFP expression maps the OVLT-SFO complex, which clearly separates into two parts–dorsal and ventral (white arrows). **(C,D)** Frontal view and confocal cross-section of *ace*^−/−^ mutant embryo. The GFP expression domain corresponding to the OVLT/SFO complex not detected (highlighted with circle). **(E,F)** Ventral view and confocal section of the *mib*^−/−^ mutant. The dorsal part of this complex is missing. **(G,H)** Ventral view and confocal section of MBL mutant, respectively. Absent of expression at region of OVLT (highlighted with circle). **(I,J)** Ventral view and confocal section of SMU mutant, respectively. Absence of OVLT domain at 48 hpf (highlighted with circle). For confocal sections, the left part represents the optical cross section and right part-saggital section. Red line through cross section indicates where saggital section is. Blue line through saggital section indicates, where cross-section is. All images are taken with 25x magnification with no zoom except *mib* and *smu* mutants, which is taken with 10x magnification with 2x zoom. Abbreviations: op, olfactory pits; ovlt, organum vasculosum of lamina terminalis. Scale bar−50 μm.

Figure S2 Transgenic zebrafish (Gateways) expresses GFP in the AP, OVLT, SFO, ME, PVO, and migratory microglia. **(A)** anti-GFP WISH and **(B)** immunohistochemistry. **(A,B)** wholemount lateral view (eyes removed); anterior to the left. ah, adenohypophysis; ap, area postrema; c, cerebellum; cpIII, choroid plexus of III ventricle; cpIV, choroid plexus of IV ventricle; d, diencephalon; e, epiphysis; h, hour postfertilization; hb, hindbrain; ht, hypothalamus; mhb, midbrain-hindbrain boundary; me, median eminence; ot, optic tectum; ovlt, organum vasculosum laminae terminalis; pt, posterior tuberculum; pvo, paraventricular organ; r5, rhombomere 5; t, telencephalon, #, SFO. Scale bar 50–μm. ^*^Defines clusters of cells expressing GFP mRNA.

Figure S3 The expression pattern of memKR in KR19 mimics that of GFP in Gateways. ap, area postrema; cp, choroid plexus; pin, pineal; rp, roof plate. Scale bar−50 μm.

Movie 1 The 3D projection of the anterior brain of the control Gateways embryo (48 hpf). The midline signal corresponds to the OVLT (ventral cluster) and the SFO (dorsal cluster). Corresponds to sections in Figures S1A,B.

Movie 2 The 3D projection of the anterior brain of the mib-/- mutant embryo on Gateways background (48 hpf). The dorsal cluster corresponding to the SFO is missing. Corresponds to sections in Figures S1E,F.
