# Supplementary figures and images for: Development of Circumventricular Organs in the Mirror of Zebrafish Enhancer-Trap Transgenics
Source: Front Neuroanat. 2017 Dec 7;11:114. doi: 10.3389/fnana.2017.00114 (PMC5770639; doi:10.3389/fnana.2017.00114)

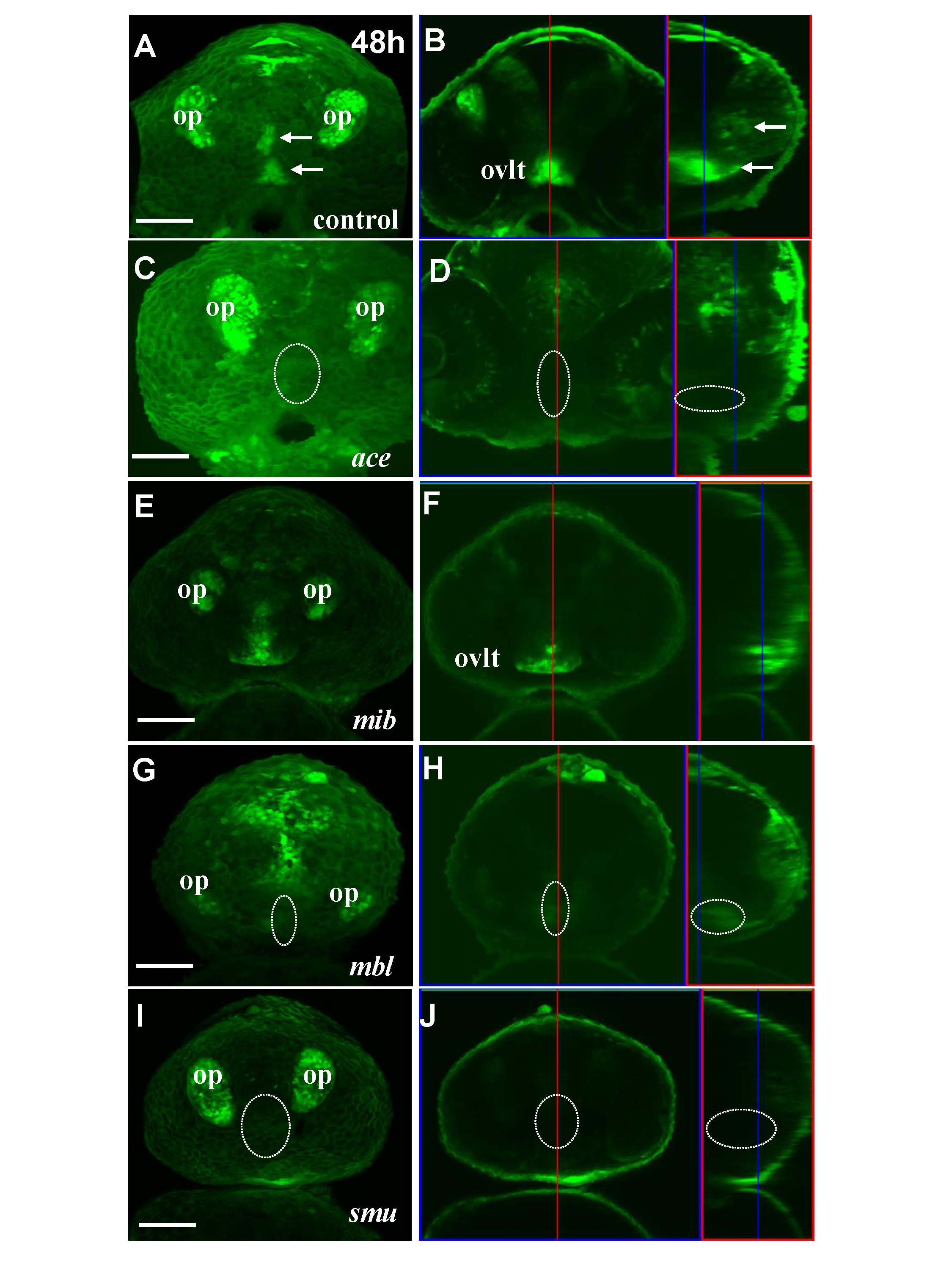

Supplement: Figure S1 — Mutant analysis of development of the OVLT and SFO at 48 hpf. (A,B) Frontal view and confocal cross-section of control embryo. Strong GFP expression maps the OVLT-SFO complex, which clearly separates into two parts–dorsal and ventral (white arrows). (C,D) Frontal view and confocal cross-section of ace−/− mutant embryo. The GFP expression domain corresponding to the OVLT/SFO complex not detected (highlighted with circle). (E,F) Ventral view and confocal section of the mib−/− mutant. The dorsal part of this complex is missing. (G,H) Ventral view and confocal section of MBL mutant, respectively. Absent of expression at region of OVLT (highlighted with circle). (I,J) Ventral view and confocal section of SMU mutant, respectively. Absence of OVLT domain at 48 hpf (highlighted with circle). For confocal sections, the left part represents the optical cross section and right part-saggital section. Red line through cross section indicates where saggital section is. Blue line through saggital section indicates, where cross-section is. All images are taken with 25x magnification with no zoom except mib and smu mutants, which is taken with 10x magnification with 2x zoom. Abbreviations: op, olfactory pits; ovlt, organum vasculosum of lamina terminalis. Scale bar−50 μm. [file Image1.JPEG]

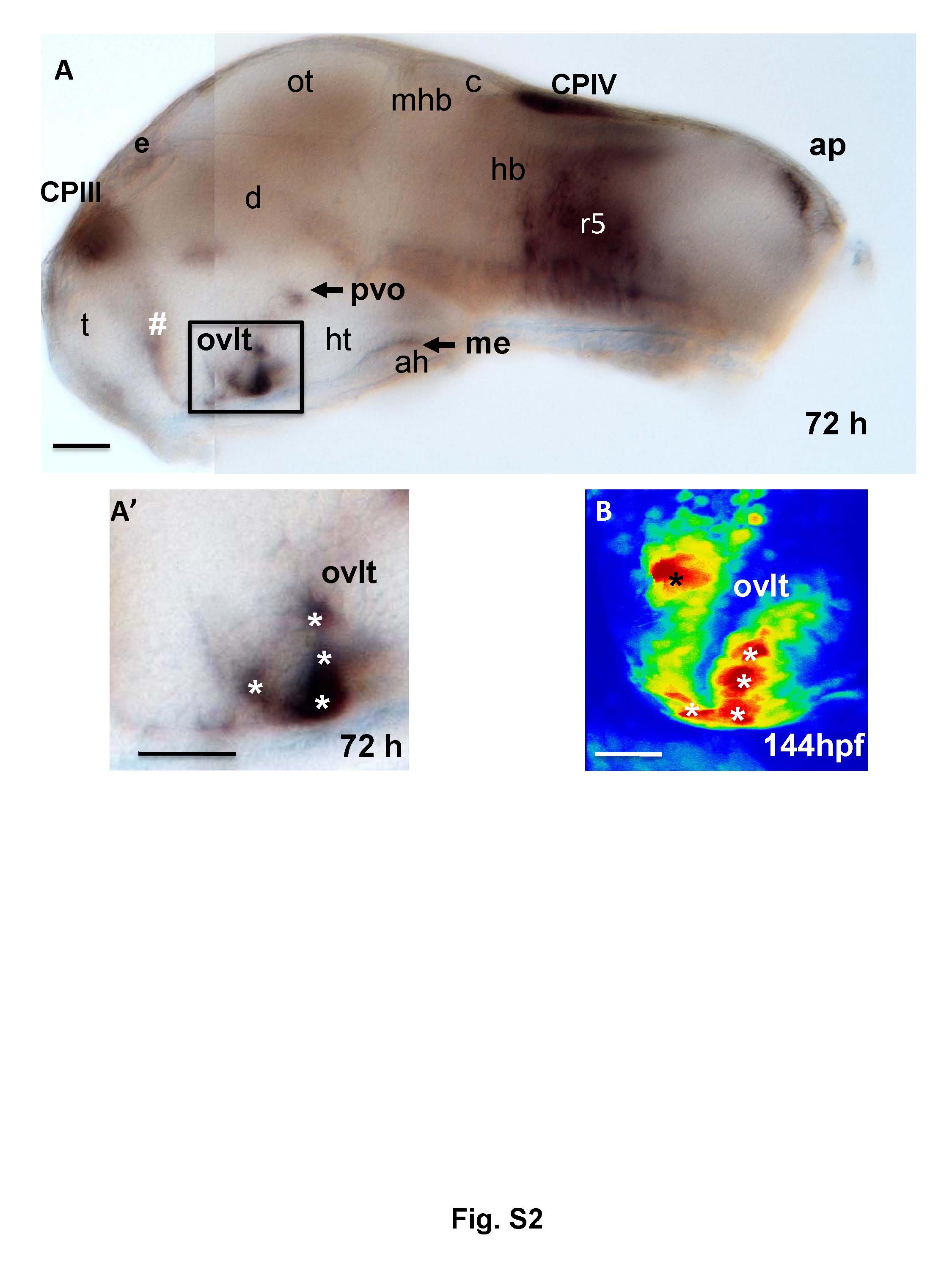

Supplement: Figure S2 — Transgenic zebrafish (Gateways) expresses GFP in the AP, OVLT, SFO, ME, PVO, and migratory microglia. (A) anti-GFP WISH and (B) immunohistochemistry. (A,B) wholemount lateral view (eyes removed); anterior to the left. ah, adenohypophysis; ap, area postrema; c, cerebellum; cpIII, choroid plexus of III ventricle; cpIV, choroid plexus of IV ventricle; d, diencephalon; e, epiphysis; h, hour postfertilization; hb, hindbrain; ht, hypothalamus; mhb, midbrain-hindbrain boundary; me, median eminence; ot, optic tectum; ovlt, organum vasculosum laminae terminalis; pt, posterior tuberculum; pvo, paraventricular organ; r5, rhombomere 5; t, telencephalon, #, SFO. Scale bar 50–μm. *Defines clusters of cells expressing GFP mRNA. [file Image2.JPEG]

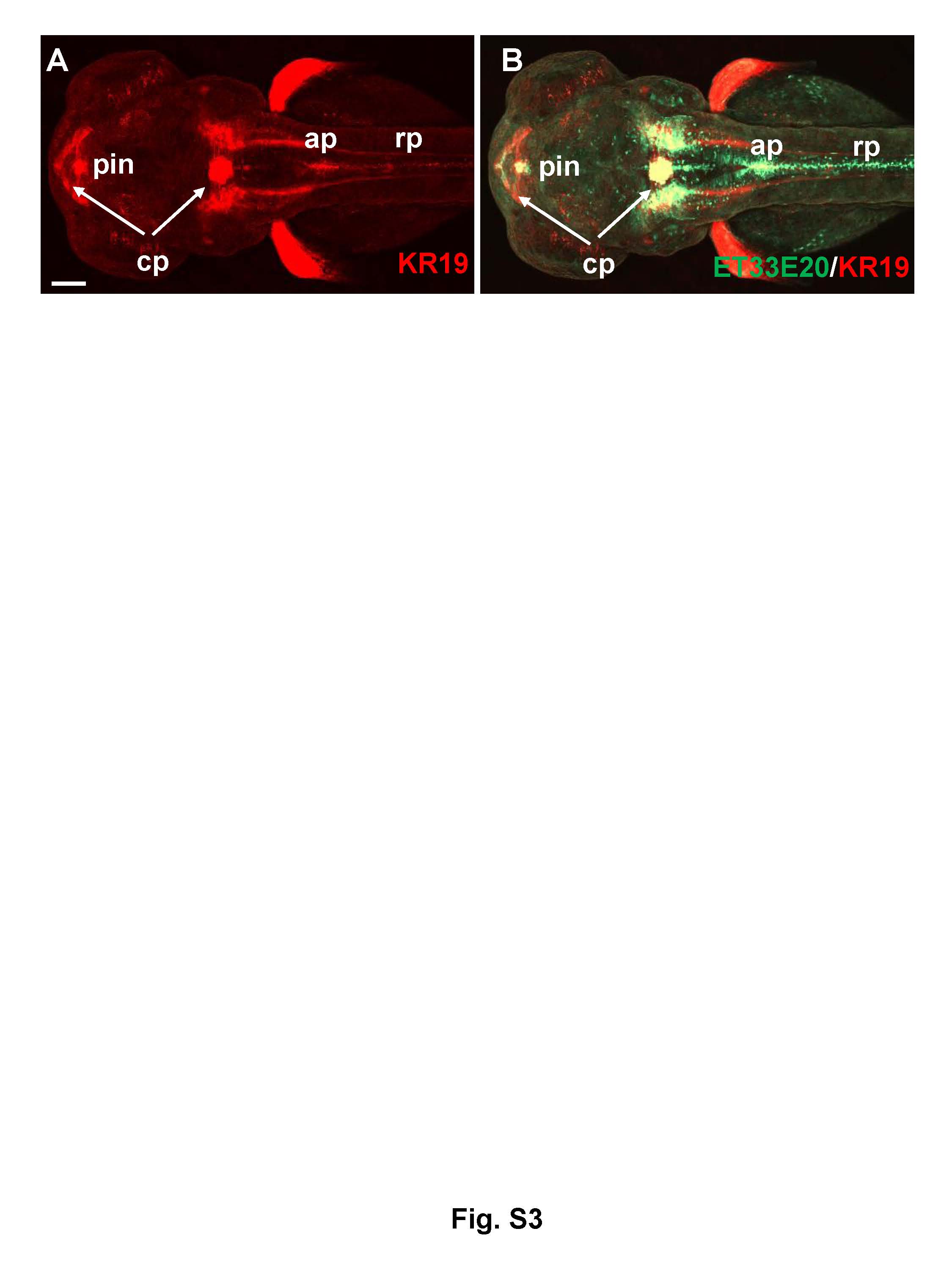

Supplement: Figure S3 — The expression pattern of memKR in KR19 mimics that of GFP in Gateways. ap, area postrema; cp, choroid plexus; pin, pineal; rp, roof plate. Scale bar−50 μm. [file Image3.JPEG]
